# Supplementary material for: DNA Ligase 4 Contributes to Cell Proliferation against DNA-PK Inhibition in MYCN-Amplified Neuroblastoma IMR32 Cells
Source: Int J Mol Sci. 2023 May 19;24(10):9012. doi: 10.3390/ijms24109012 (PMC10219512; doi:10.3390/ijms24109012)
Supplement: Supplementary file 1 [file ijms-24-09012-s001.zip › ijms-2369278-supplementary.pdf]

Materials and Methods

ATP assays for cell viability

Cells were diluted to 10,000 cells/100 µL and seeded into each well of a 96-well plate. After incubation for the indicated times and conditions, 100 µL CellTiter-Glo 2.0 Reagent (Promega, Madison, WI, USA) was added to the cells. After 2 min with gentle agitation, cells were incubated for 10min at RT, and the luminescence was measured using an infinite F200 PRO plate reader (TECAN, Männedorf, Zurich, Switzerland).

RNA Interference

Silencer Select Validated siRNA specific for LIG4 (s8181) and a negative control were obtained from ambion (Carlsbad,CA, USA). Cells were transiently transfected with 50 nM siRNA using Lipofectamine RNAiMAX (Invitrogen) reagent and were prepared 24 h after transfection for each experiment. Knockdown efficiency of siRNA was measured by quantitative reverse-transcription PCR at 72h after transfection.

Supplementary Figures

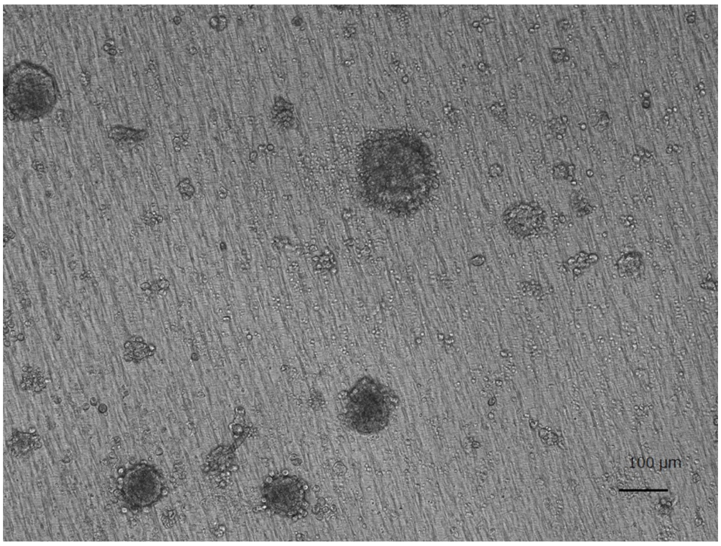

Figure S1. An image of abdominal neuroblastoma spheroids from MYCN homozygotes. Spheroids were seeded on VECELL 24 well GAS PERMEABLE Plate (Cosmo Bio, Tokyo, Japan.) for overnight, and the phase contrast image was taken by BZ-X710 (Keyence, Osaka, Japan)

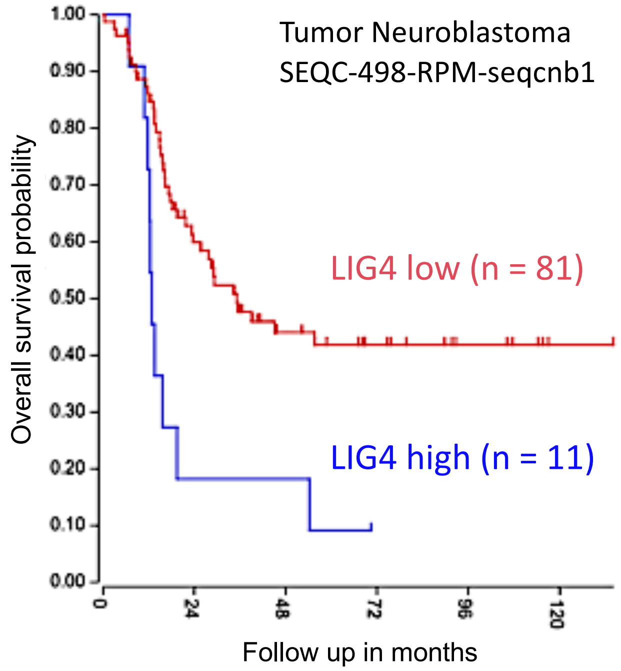

Figure S2. Kaplan–Meier survival curves of 92 patients with MYCN-amplified neuroblastoma stratified by high or low LIG4 mRNA expression (Tumour Neuroblastoma SEQC-498-RPM-seqcnb1, raw P = 0.0025 and Bonferroni corrected P = 0.194).

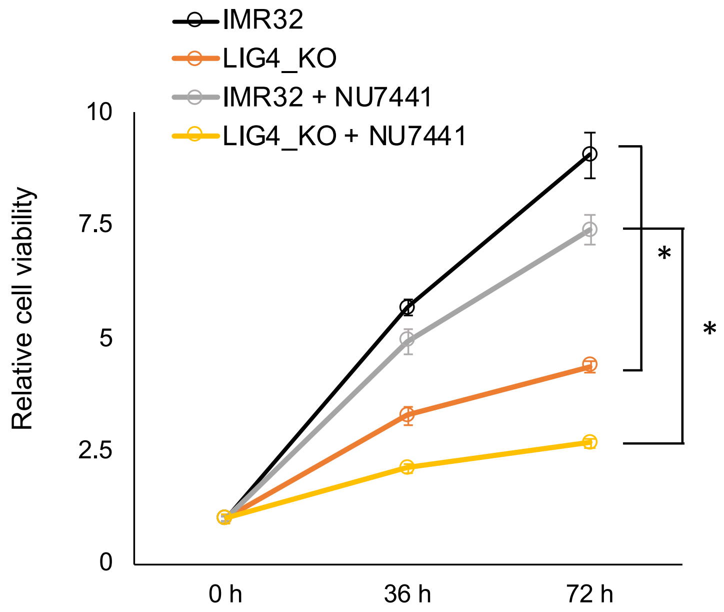

Figure S3. An ATP assay in IMR32 cells, in which LIG4 is ablated or not (LIG4_KO or IMR32, respectively) after treatment with or without 3 μM NU7441 for the indicated time course. Relative cell viability is normalised by those of corresponding untreated cells. Statistical significance is presented as the mean ± standard deviation (SD) of triplicates. *P <0.01.

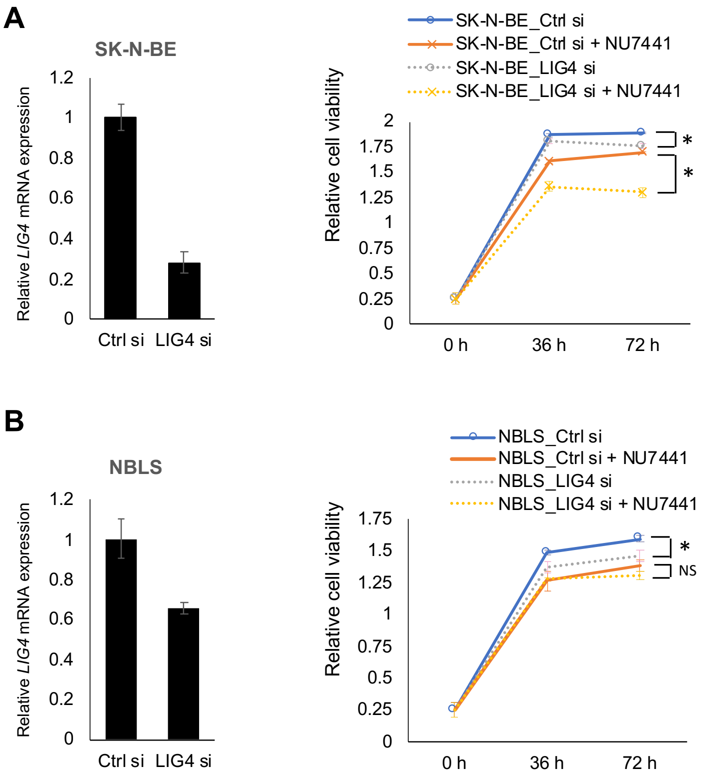

Figure S4. Efficiency of siRNA-mediated LIG4 knockdown (left panel) and cell viability assays (right panel) in (A) SK-N-BE and (B) NBLS cells, in which LIG4 siRNA or negative control siRNA are transfected (LIG4 si or Ctrl si, respectively). *LIG4* mRNA is measured by quantitative reverse-transcription PCR. After treatment with or without 3 μM NU7441, Alamar Blue dye (resazurin) reduction are measured at the indicated time points. Relative cell viability is normalised by those of corresponding untreated cells. Statistical significance is presented as the mean ± standard deviation (SD) of triplicates. *P <0.01. NS, not significant.

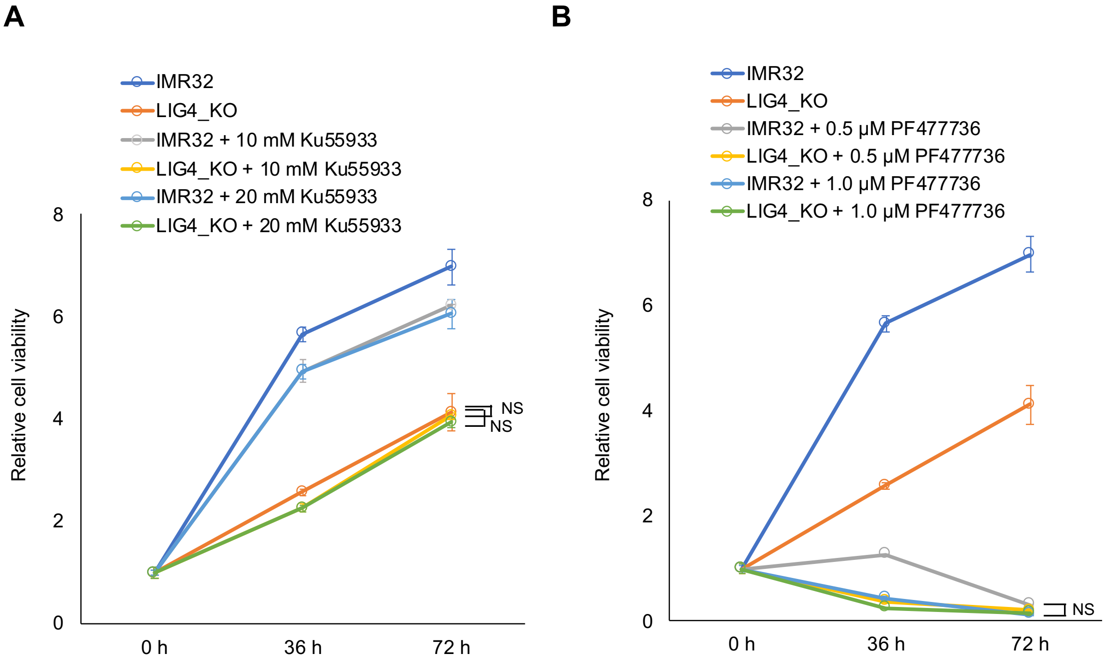

Figure S5. Cell viability assay in IMR32 cells, in which LIG4 is ablated or not (LIG4_KO or IMR32, respectively) after treatment with or without (A) 10 or 20 mM Ku55933 (ATM inhibitor), or (B) 0.5 or 1.0 μM PF477736 (CHK1 inhibitor) for the indicated time course. Relative cell viability is measured as Alamar Blue dye (resazurin) reduction, and normalised by those of corresponding untreated cells. Statistical significance is presented as the mean ± standard deviation (SD) of triplicates. *P <0.01. NS, not significant.
